# Diffuse Large B-Cell Lymphoma of the Ethmoid Sinus With Orbital Invasion: A Case Report

**DOI:** 10.7759/cureus.109207

**Published:** 2026-05-19

**Authors:** Yahya Boualam, Achraf Sbai, Drissia Benfadil, Azzedine Lachkar

**Affiliations:** 1 Department of Otolaryngology - Head and Neck Surgery, Faculty of Medicine and Pharmacy, Mohammed First University of Oujda, Oujda, MAR; 2 Department of Otolaryngology - Head and Neck Surgery, Mohammed VI University Hospital, Oujda, MAR

**Keywords:** ann arbor staging system, chemotherapy, diffuse large b-cell lymphoma, orbital invasion, sinonasal lymphoma

## Abstract

Diffuse large B-cell lymphoma (DLBCL) rarely involves the paranasal sinuses, and ethmoidal localization with orbital extension is exceptional. We report the case of a 76-year-old patient presenting with progressive nasal obstruction and epiphora. Imaging revealed an ethmoidal mass with orbital invasion. Histopathological examination confirmed the diagnosis of DLBCL.

The patient was referred to the medical oncology department for systemic immunochemotherapy followed by consolidative radiotherapy. No surgical intervention was performed.

This case emphasizes the need to consider lymphoma in the differential diagnosis of aggressive sinonasal masses with orbital involvement. Early diagnosis and appropriate oncologic management are crucial for optimal outcomes.

## Introduction

Diffuse large B-cell lymphoma (DLBCL) is the most common germinal center-derived malignant lymphoma and accounts for nearly 30%-40% of non-Hodgkin lymphoma (NHL) cases in adults globally. It represents a heterogeneous group of disorders with variable clinical outcomes, defined by differences in clinical presentation, cell of origin (COO), molecular characteristics, and recurrent genetic alterations [1,2].

Primary sinonasal lymphoma is a rare extranodal manifestation of non-Hodgkin lymphoma, accounting for roughly 0.17% of all lymphoma cases. This entity has a distinct clinical behavior compared with other lymphomas and often presents a diagnostic challenge [1,3].

Clinical presentation is usually nonspecific, often leading to confusion with benign conditions such as rhinosinusitis [4,5]. Clinical manifestations vary and are numerous due to the important and complex anatomical relationships of the region, particularly with the orbit, nasal cavities, and neurovascular structures. Reported symptoms include headache, facial pain, nasal obstruction, and visual disturbances, often contributing to delays in accurate diagnosis and timely treatment [6].

## Case presentation

The patient was a 76-year-old former carpenter with a history of hypertension treated with calcium channel blockers and a 42-year history of smoking. He presented with progressively worsening nasal obstruction over approximately one year, associated with left-sided epiphora for the past three months.

Clinical examination revealed nodular lesions and inflammation at the medial canthus of the left eye, accompanied by epiphora (Figure 1). Anterior rhinoscopy demonstrated a tissue mass completely occupying the left nasal cavity and deviating the nasal septum to the right (Figure 2). Endoscopic examination of the right nasal cavity showed a budding mass involving the middle portion of the nasal cavity, particularly the middle turbinate.

**Figure 1:**
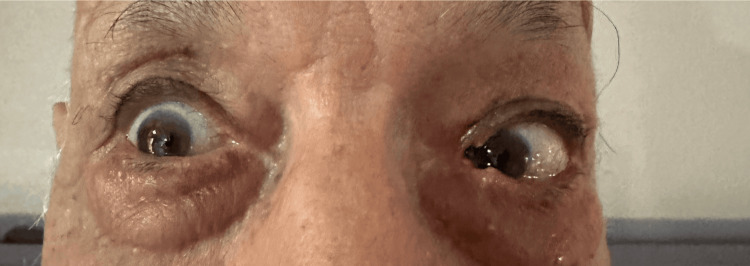
Clinical photograph showing a lesion involving the medial canthus associated with left eye epiphora

**Figure 2:**
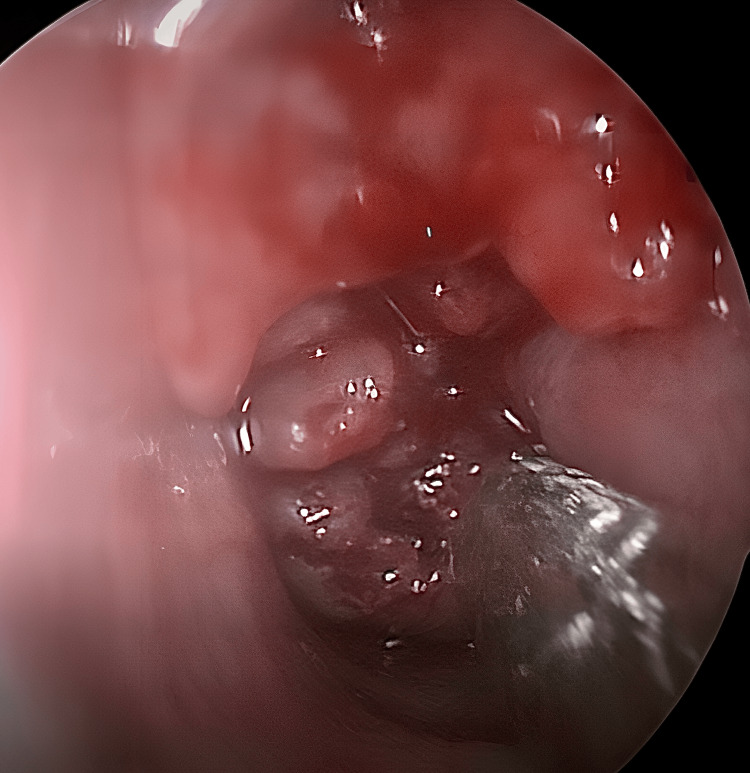
Image of anterior rhinoscopy (during the biopsy procedure) demonstrating a tissue mass completely occupying the left nasal cavity and deviating the nasal septum to the right

Palpation of the cervical, axillary, inguinal, and femoral lymph node regions revealed no lymphadenopathy.

A computed tomography (CT) scan of the sinonasal region demonstrated a large soft tissue mass in the left nasal cavity extending into the ethmoid and maxillary sinuses, with obstruction of the frontal sinus. There was destruction of the ethmoidal septa, the medial wall of the maxillary sinus, and the lamina papyracea, with extension into the left orbit. The nasal septum was also involved, with tumor extension into the contralateral nasal cavity (Figure 3).

**Figure 3:**
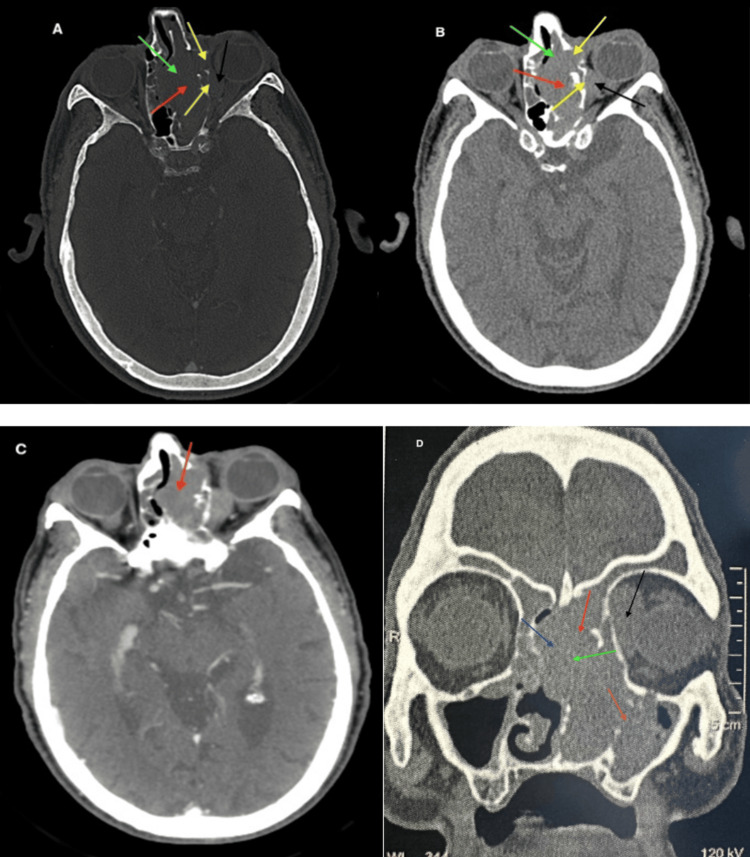
Paranasal sinus CT scan at presentation (A) Axial bone window showing a left ethmoidal mass (red arrow) eroding the lamina papyracea (yellow arrow) with orbital invasion (black arrow), along with involvement of the nasal septum and extension into the right nasal cavity (green arrow). (B) Axial parenchymal window demonstrating the same findings as in panel A. (C) Contrast-enhanced axial parenchymal image showing the ethmoidal mass (red arrow) eroding the ethmoidal septa, lamina papyracea, and bony nasal septum, with invasion of the orbit and medial rectus muscle, reaching the globe and the optic nerve. (D) Coronal parenchymal image demonstrating a large mass occupying the entire left nasal cavity, extending into the orbit and ipsilateral maxillary sinus, with destruction of the nasal septum and extension into the right nasal cavity. CT: computed tomography

A thoracoabdominopelvic CT scan was performed and showed no evidence of distant involvement. Lactate dehydrogenase (LDH) levels were within normal limits. Bone marrow evaluation was not performed because there were no cytopenias, systemic symptoms, or other clinical or laboratory findings suggestive of bone marrow involvement.

According to the Ann Arbor staging system, the disease was classified as stage IE because it was confined to the sinonasal region without nodal or distant involvement.

Given the patient’s occupational exposure, age, ethmoidal location of the lesion, and the presence of bone destruction, ethmoidal adenocarcinoma was initially suspected, followed by squamous cell carcinoma, particularly in the context of a history of smoking.

A biopsy performed under anterior rhinoscopy was consistent with diffuse large B-cell lymphoma (DLBCL), as confirmed by immunohistochemistry, which showed positivity for CD20, BCL2, CD10, and BCL6, with a Ki-67 proliferation index estimated at 50% (Figure 4).

**Figure 4:**
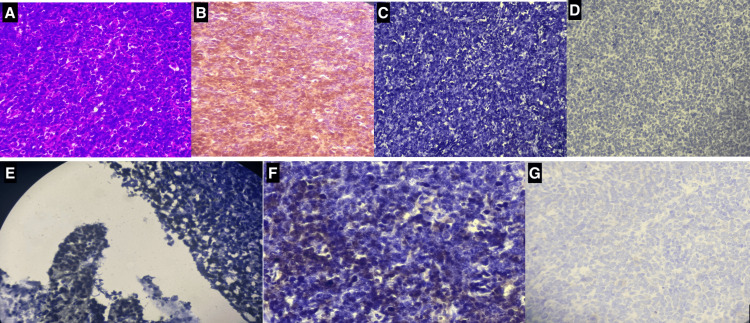
Histopathological and immunohistochemical findings (A) Histological sections show large mononuclear cells with ovoid to irregular nuclei, dispersed chromatin, prominent nucleoli, and scant cytoplasm. (B) The tumor cells show strong positivity for CD20 on immunohistochemistry. (C) CD5 is negative. (D) Cyclin D1 is negative. (E) CD10 is positive. (F) BCL6 is positive. (G) Ki-67 proliferation index is approximately 50%.

The lymphoma was classified as a germinal center B-cell (GCB) subtype owing to the positivity of CD10 and BCL6 markers.

The patient was referred to the medical oncology department, and he received three cycles of rituximab, cyclophosphamide, doxorubicin, vincristine, and prednisolone (R-CHOP) chemotherapy, followed by consolidative radiotherapy.

The treatment was generally well tolerated. The patient experienced mild fatigue and transient hematological toxicity without febrile neutropenia or severe infectious complications.

Complete resolution on follow-up CT imaging was achieved after nine months of treatment. The patient was subsequently monitored in our department with regular clinical and nasofibroscopic examinations. During a five-year follow-up period, no evidence of recurrence was observed. The patient and his family were advised to seek prompt medical attention in the event of any new symptoms.

## Discussion

Non-Hodgkin lymphomas (NHLs) are among the most common malignancies in developed countries, ranking fourth in incidence and accounting for approximately 3%-5% of cancer-related deaths and nearly 1% of all-cause mortality [7]. Extranodal involvement occurs in approximately 10%-58% of non-Hodgkin lymphomas (NHLs). Sinonasal DLBCLs are rare, representing nearly 3% of head and neck neoplasms. This sinonasal localization is particularly uncommon, accounting for only about 0.17% of all lymphomas. From a histological perspective, B-cell lymphomas predominate, while T-cell lymphomas are rarely encountered [8].

Diffuse large B-cell lymphoma (DLBCL) is the most common form of non-Hodgkin lymphoma worldwide [9,10]. It primarily affects elderly patients, with a median age at diagnosis in the seventh decade, although cases may also occur in young adults and, less frequently, in children. The disease shows a slight predominance in men [11].

Diffuse large B-cell lymphoma (DLBCL) represents a biologically heterogeneous entity at the clinical, genetic, and molecular levels, comprising multiple subtypes associated with diverse molecular abnormalities involving genetic, protein, and microRNA pathways [12].

The tumor cells expressed CD10 and BCL6, supporting a germinal center B-cell (GCB) phenotype according to the Hans algorithm. This subtype of diffuse large B-cell lymphoma is generally associated with a more favorable prognosis compared with the non-germinal center subtype [13].

Multiple recognized or suspected predisposing factors have been reported, such as viral infections, immunosuppression, exposure to environmental toxins, bacterial infections, and tobacco use [14].

Clinical symptoms are typically nonspecific and often appear relatively mild compared with the extent of the tumor [15]. Systemic manifestations are uncommon, whereas local symptoms are typically dominated by long-standing nasal obstruction, as observed in our patient, occasionally accompanied by rhinorrhea or recurrent epistaxis [15].

Orbital invasion in sinonasal DLBCL has historically been associated with locally aggressive disease and an increased risk of central nervous system (CNS) extension. However, outcomes have significantly improved in the rituximab era, with a favorable prognosis reported after appropriate chemoimmunotherapy [16].

Radiographic features generally show bulky lesions with an infiltrative growth pattern, often resulting in prominent erosion of the sinus walls [17,18].

Imaging studies may reveal a well-defined mass or, less frequently, opacification of the sinuses on radiographs [19]. High-grade B-cell tumors have been reported to cause destruction of the orbital soft tissues and adjacent bone structures, while perforation or destruction of the nasal septum is more frequently linked to high-grade T-cell lymphomas [18].

Computed tomography (CT) plays a crucial role in assessing the extent of structural destruction and the involvement of the sinonasal tract and orbits, and is considered the reference imaging modality [19]. MRI may help assess locoregional extension and differentiate neoplastic from inflammatory lesions, while definitive diagnosis relies on histopathological and immunohistochemical examination.

Patients presenting with sinonasal lymphomas tend to have a better prognosis than those with nodal lymphomas of comparable histological grades [17].

Sinonasal squamous cell carcinoma may constitute a true differential diagnosis of sinonasal DLBCL on imaging. However, lymphoma typically appears as a more homogeneous soft tissue mass with relatively limited bone destruction, making histopathological examination essential for definitive diagnosis.

The most common treatment for diffuse large B-cell lymphoma of the sinonasal tract is still chemotherapy. The role of surgical intervention remains limited, as its benefit in improving tumor control or survival has not been clearly demonstrated [17].

In the study by Boussen et al. [20], therapeutic outcomes were markedly superior in patients with localized disease. While the overall complete response rate was 35%, patients with stage IE lymphoma and low-grade tumors achieved a complete remission rate of 100%. Similarly, the five-year overall survival reached 92% in patients with stage IE lymphoma confined to the nasal cavity, compared with 69% in the overall population and only 33% in advanced-stage or high-grade disease (Table 1). The favorable therapeutic outcome observed in our patient is consistent with these findings, with complete remission achieved in stage IE disease.

**Table 1:** Therapeutic outcomes of the series reported by Boussen et al. (ENT Department, Charles Nicolle Hospital, Tunis) PD: progressive disease, ENT: ear, nose, and throat Source: [20]

Response	Number of patients (%)
Complete response	8 (35%), stage IE: 100%, low grade: 100%
Partial response	3 (13%), high grade: 2
Failure	12 (52%), PD: 2, metastases: 3, deaths: 7

## Conclusions

Sinonasal diffuse large B-cell lymphoma with orbital invasion is a rare and potentially misleading presentation that may mimic other aggressive inflammatory or neoplastic conditions. This case highlights the importance of maintaining a high index of suspicion when evaluating rapidly progressive ethmoidal masses with orbital extension. Early histopathological confirmation is essential to establish the diagnosis and avoid unnecessary surgical procedures. Prompt referral to oncology and initiation of systemic immunochemotherapy remain the cornerstone of management and are associated with favorable outcomes.
